# Supplementary material for: Practitioner training and user experience of Seeking Safety for people with complex post-traumatic stress disorder and substance use disorder
Source: PeerJ. 2025 Sep 22;13:e20010. doi: 10.7717/peerj.20010 (PMC12462685; doi:10.7717/peerj.20010)
Supplement: Supplemental Information 1 [file peerj-13-20010-s001.pdf]

SECTION	ITEM	PRISMA-ScR CHECKLIST ITEM	REPORTED ON PAGE #
**TITLE**			
Title	1	Identify the report as a scoping review.	1
**ABSTRACT**			
Structured summary	2	Provide a structured summary that includes (as applicable): background, objectives, eligibility criteria, sources of evidence, charting methods, results, and conclusions that relate to the review questions and objectives.	1
**INTRODUCTION**			
Rationale	3	Describe the rationale for the review in the context of what is already known. Explain why the review questions/objectives lend themselves to a scoping review approach.	4
Objectives	4	Provide an explicit statement of the questions and objectives being addressed with reference to their key elements (e.g., population or participants, concepts, and context) or other relevant key elements used to conceptualize the review questions and/or objectives.	5
**METHODS**			
Protocol and registration	5	Indicate whether a review protocol exists; state if and where it can be accessed (e.g., a Web address); and if available, provide registration information, including the registration number.	5
Eligibility criteria	6	Specify characteristics of the sources of evidence used as eligibility criteria (e.g., years considered, language, and publication status), and provide a rationale.	6
Information sources*	7	Describe all information sources in the search (e.g., databases with dates of coverage and contact with authors to identify additional sources), as well as the date the most recent search was executed.	6
Search	8	Present the full electronic search strategy for at least 1 database, including any limits used, such that it could be repeated.	6
Selection of sources of evidence†	9	State the process for selecting sources of evidence (i.e., screening and eligibility) included in the scoping review.	6
Data charting process‡	10	Describe the methods of charting data from the included sources of evidence (e.g., calibrated forms or forms that have been tested by the team before their use, and whether data charting was done independently or in duplicate) and any processes for obtaining and confirming data from investigators.	7
Data items	11	List and define all variables for which data were sought and any assumptions and simplifications made.	7
Critical appraisal of individual sources of evidence§	12	If done, provide a rationale for conducting a critical appraisal of included sources of evidence; describe the methods used and how this information was used in any data synthesis (if appropriate).	7

SECTION	ITEM	PRISMA-ScR CHECKLIST ITEM	REPORTED ON PAGE #
Synthesis of results	13	Describe the methods of handling and summarizing the data that were charted.	7
**RESULTS**			
Selection of sources of evidence	14	Give numbers of sources of evidence screened, assessed for eligibility, and included in the review, with reasons for exclusions at each stage, ideally using a flow diagram.	7
Characteristics of sources of evidence	15	For each source of evidence, present characteristics for which data were charted and provide the citations.	7
Critical appraisal within sources of evidence	16	If done, present data on critical appraisal of included sources of evidence (see item 12).	7
Results of individual sources of evidence	17	For each included source of evidence, present the relevant data that were charted that relate to the review questions and objectives.	7
Synthesis of results	18	Summarize and/or present the charting results as they relate to the review questions and objectives.	8
**DISCUSSION**			
Summary of evidence	19	Summarize the main results (including an overview of concepts, themes, and types of evidence available), link to the review questions and objectives, and consider the relevance to key groups.	9
Limitations	20	Discuss the limitations of the scoping review process.	13
Conclusions	21	Provide a general interpretation of the results with respect to the review questions and objectives, as well as potential implications and/or next steps.	14
**FUNDING**			
Funding	22	Describe sources of funding for the included sources of evidence, as well as sources of funding for the scoping review. Describe the role of the funders of the scoping review.	15

JBI = Joanna Briggs Institute; PRISMA-ScR = Preferred Reporting Items for Systematic reviews and Meta-Analyses extension for Scoping Reviews.
* Where *sources of evidence* (see second footnote) are compiled from, such as bibliographic databases, social media platforms, and Web sites.
† A more inclusive/heterogeneous term used to account for the different types of evidence or data sources (e.g., quantitative and/or qualitative research, expert opinion, and policy documents) that may be eligible in a scoping review as opposed to only studies. This is not to be confused with *information sources* (see first footnote).
‡ The frameworks by Arksey and O'Malley (6) and Levac and colleagues (7) and the JBI guidance (4, 5) refer to the process of data extraction in a scoping review as data charting.
§ The process of systematically examining research evidence to assess its validity, results, and relevance before using it to inform a decision. This term is used for items 12 and 19 instead of "risk of bias" (which is more applicable to systematic reviews of interventions) to include and acknowledge the various sources of evidence that may be used in a scoping review (e.g., quantitative and/or qualitative research, expert opinion, and policy document).

From: Tricco AC, Lillie E, Zarin W, O'Brien KK, Colquhoun H, Levac D, et al. PRISMA Extension for Scoping Reviews (PRISMA-ScR): Checklist and Explanation. Ann Intern Med. ;169:467–473. doi: 10.7326/M18-0850

Supplementary File: Table 1: Search strategy for PuBMed and study inclusion criteria

Study selection criteria (PICOTS)		
	**Inclusion criteria**	**Exclusion criteria**
**Population**	• Critically ill children admitted to a Paediatric Intensive Care unit birth – 18 years of age	• Children > 18 years of age<br>• Infants < 37 weeks age
**Intervention**	• Any study describing micronutrient status or supplementation in critically ill children<br>• Vitamins and trace elements: vitamin A, vitamin C (ascorbic acid), thiamine (vitamin B1), vitamin B12, vitamin B6 (pyridoxine), niacin (vitamin B3), folic acid, manganese, molybdenum, zinc, selenium, copper, iodine, iron, molybdenum, chromium, pantothenic acid, riboflavin (vitamin B2), biotin, manganese, vitamin E (α-tocopherol), β-carotene	• Any study describing micronutrient status or supplementation in children who are not critically ill requiring admission to intensive care<br>• Electrolytes and minerals; calcium, magnesium, potassium, phosphate, sodium, vitamin D
**Comparison**	• Any study involving micronutrient supplement or those characterising serum levels in critically ill children	• Adults, pre-term infants, non-critically ill children, inherited metabolic disorders
**Outcome**	• Serum levels of micronutrients in critically ill children<br>• Studies reporting serum levels associated with a defined outcome measure e.g. paediatric intensive care unit length of stay, duration of mechanical venital, mortality<br>• Studies considering micronutrient supplementation and association with defined outcomes measures	• Genome wide association, in-vitro, in-vivo, animal studies, those studies not considering serum micronutrient levels
**Timing**	• Acute, stable phase of critical illness e.g. paediatric care admission period<br>• Recovery or rehabilitation e.g. post-discharge from paediatric intensive care	• Recovery or rehabilitation not following an intensive care unit admission<br>• Community or general ward patient
**Setting**	• Paediatric Intensive care	• In patient – hospital ward, community setting
**Search strategy**		
**Search words**	• key terms: “paediatric intensive care” (paediatric critical care OR paediatric critical illness OR critically ill) AND “micronutrients” (vitamins	

	OR trace elements OR vitamin D OR vitamin A OR β-carotene OR vitamin C OR ascorbic acid OR thiamine OR vitamins B12 OR vitamin B6 OR pyridoxine OR niacin OR folic acid vitamin E Orα-tocopherol OR manganese OR molybdenum OR zinc OR selenium OR copper OR iodine OR molybdenum OR chromium OR pantothenic acid OR riboflavin OR biotin) AND "children" AND (infants OR paediatric) AND recovery (OR rehabilitation) which were adapted for searching each database<br>• Backwards and forwards searches were completed<br>• A search was completed for each named vitamin or trace element combined with "paediatric intensive care" and "children"
**Limits**	• Filters: Humans; English; Child: birth-18 years
**Year range**	• No limits – up to 3rd August 2019
**Publication type**	• English language
**Search example: PUBMED**	• (((((micronutrients AND (vitamins OR trace elements)))) AND ((children AND (infants or paediatric)))) AND ((paediatric critical illness and (critically ill children or paediatric intensive care))) AND recovery (((OR rehabilitation)))))) Filters: Humans; English; Child: birth-18 years
**Search results**	
**Stage 1: Identification**	• 6 databases, 68 searches, unpublished literature, reference list cross – checking, internet search (n=694)
**Stage 2: 1st screening**	• Screened and duplicates removed (n=361)
**Stage 3: 2nd screening – eligibility**	• Second details review assessing inclusion and exclusion criteria (abstract information) (n=88)
**Stage 4: Data extraction**	• Data extraction, further review excluding non-ICU related studies and recheck inclusion criteria (n=84)
**Stage 5: Collating, summarising and reporting the results**	• 1st: Charting of study title, authors, year, duration of study, study design, objectives, study numbers, methods, results including continuous and categorical variables, and key findings<br>• 2nd: Content analysis was completed by selecting, coding and creating initial codes, sub-categories and overarching themes/categories
