# Supplementary material for: Practitioner training and user experience of Seeking Safety for people with complex post-traumatic stress disorder and substance use disorder
Source: PeerJ. 2025 Sep 22;13:e20010. doi: 10.7717/peerj.20010 (PMC12462685; doi:10.7717/peerj.20010)
Supplement: Supplemental Information 2 [file peerj-13-20010-s002.docx]

| **Initial coding (n=113)** | **Sub-categories (n=11)** | **Overarching themes (n=5)** |
| --- | --- | --- |
| Baseline to 6-month and 9-month follow-up | Content of sessions  Number of sessions  Standardisation | Seeking Safety as a treatment |
| Baseline to end of treatment |  |  |
| Baseline to three month follow up |  |  |
| Incentives |  |  |
| Integrative treatments as a potential mediator towards an endpoint vs. mechanism of action |  |  |
| Modelling group membership turnover |  |  |
| Novel methods for mediation analysis |  |  |
| Present focused coping skills approach |  |  |
| Seeking safety check-in |  |  |
| Seeking safety manualized therapy |  |  |
| Sequential process effect |  |  |
| Session components |  |  |
| Session length |  |  |
| Standard protocol for 25 topics/ sessions |  |  |
| Standardisation |  |  |
| Trauma informed |  |  |
| Trauma narration not required |  |  |
| Hard to reach and under-represented groups | Ideal group composition | Meeting the needs of a diverse patient population group |
| Men only |  |  |
| Mixed gender |  |  |
| Seeking safety – females vs. males |  |  |
| Transgender |  |  |
| Women only |  |  |
| Clinician competence | Interplay between PTSD and SUD  Treatment fidelity - sessions and attendance | Factors impacting success |
| Continued gains end of treatment to follow up |  |  |
| Delayed emergence effect |  |  |
| Depressive symptoms |  |  |
| Differences between high – moderate – low attenders |  |  |
| Discordance with substance abuse |  |  |
| Duration of sessions |  |  |
| Flexibility in the number of sessions attended |  |  |
| Group facilitator as safety conductor |  |  |
| Helpfulness – first to last – topic, therapist, handout and quotation |  |  |
| Impediments to recovery |  |  |
| Improvement, implementation and treatment fidelity measures |  |  |
| Improvements over time |  |  |
| Interplay between PTSD and SUD |  |  |
| Length of stay |  |  |
| Low literacy levels – impact effectiveness |  |  |
| Minimum dose required |  |  |
| One-on-one exit interviews |  |  |
| Peer led Seeking Safety groups |  |  |
| Preparedness and capacity to commit |  |  |
| Present focused coping skills approach |  |  |
| Readiness to commit to the group |  |  |
| Social support and solidarity |  |  |
| Timing/ context of intervention |  |  |
| Treatment adherence |  |  |
| Treatment satisfaction |  |  |
| Treatment services review |  |  |
| Usefulness of treatments |  |  |
| Not discussing the trauma | Agency over self  Group - social support & validation | Empowerment of self and agency over life |
| Action to prevent drug use and control PTSD |  |  |
| Attending to needs of SUD disorder first |  |  |
| Avoidance of discussion past trauma |  |  |
| Awareness building - insight/ reflection |  |  |
| Basic education on PTSD and SUD |  |  |
| Better understanding of self and the role of substances |  |  |
| Challenges to safety and feeling unsafe in a group |  |  |
| Chronic painful life histories |  |  |
| Cognitive restructuring – maladaptive thoughts associated with substance use and trauma |  |  |
| Concurrent treatment |  |  |
| Connection with group as a source of support and validation |  |  |
| Detaching from emotional pain |  |  |
| Developing and strengthening foundations of self |  |  |
| Developing effective communication skills to build a healthy support network |  |  |
| Different view on life |  |  |
| Discussing the trauma |  |  |
| Drug use disorder (i.e. nicotine, cocaine, marijuana) |  |  |
| Evidence based past-focused vs. present focused |  |  |
| Evoking emotions |  |  |
| Feeling empowered to exert agency over life |  |  |
| Focus on relationship issues |  |  |
| Focus on the present |  |  |
| Goals of reducing substance abuse |  |  |
| Guilt not being a central focus |  |  |
| Honesty and community resources |  |  |
| Lifetime traumas |  |  |
| Maladaptive coping |  |  |
| Negative impact of trauma |  |  |
| Safety and feeling validated |  |  |
| Seeking safety is not an island |  |  |
| Substance dependence |  |  |
| Taking good care of yourself |  |  |
| Among those who do improve as a function of treatment, do different treatment pathways lead to subsequent improvement and remission of symptoms? | What are the mechanisms of action and what leads to sustained remission?  What training is required to successfully deliver the treatment?  What, how, when, and who would benefit from Seeking safety treatment? | Knowledge gaps around Seeking Safety treatment |
| Are differences in outcomes due to sociodemographic or clinical characteristics? |  |  |
| Are Peer-Led Seeking Safety groups effective? |  |  |
| Are Peer-Led Seeking Safety groups sustainable? |  |  |
| Are there particular patient and clinician characteristics that predict who does best with seeking safety? |  |  |
| Are treatment topics essential? |  |  |
| Calls to advance in the methodology used to evaluate treatment interventions for comorbid PTSD and SUD? |  |  |
| Calls to examination of mechanisms of action in the treatment of co-morbid PTSD and SUD |  |  |
| Do all patient subgroups benefit equally? |  |  |
| Does participant evaluation of therapist alongside treatment retention, outcome and satisfaction date influence treatment outcomes? |  |  |
| How can we understand the finding that relapse prevention, a standard substance abuse treatment, equally reduces PTSD symptoms? |  |  |
| How do the two treatments compare in the emotional intensity? |  |  |
| How does it fare in group versus individual modality? |  |  |
| How is Seeking Safety different from regular treatments? |  |  |
| Is it possible to track symptom changes are each group to provide precise information about the minimum effective dose? |  |  |
| Is the reduction in PTSD symptoms due to effects of the integrative treatment in mediating an intermediate endpoint or as a mechanism of treatment action? |  |  |
| Is there an added benefit in conducting Seeking Safety first and then Creating Change? |  |  |
| What are the changes to SUD over time? |  |  |
| What are the characteristics of therapist and settings might promote successful Creating Change outcomes? |  |  |
| What are the mechanisms of action of each treatment? |  |  |
| What are the most effective group mixed-groups or split-gender groups? |  |  |
| What is the causal ordering of the relationship between PTSD and SUD? |  |  |
| What is the dose required, number of sessions and topics? |  |  |
| What is the ideal follow up time-period? |  |  |
| What models of treatment appeal and is feasible to patients, therapists, and treatment programmes? |  |  |
| What training is necessary? |  |  |
| Which clients are most likely to benefit for each treatment? |  |  |
| Which symptoms are quicker to change? |  |  |
| Would simultaneous treatment of trauma related disorders be beneficial for SUD or a sequential model? |  |  |
