# Supplementary material for: Practitioner training and user experience of Seeking Safety for people with complex post-traumatic stress disorder and substance use disorder
Source: PeerJ. 2025 Sep 22;13:e20010. doi: 10.7717/peerj.20010 (PMC12462685; doi:10.7717/peerj.20010)
Supplement: Supplemental Information 3 [file peerj-13-20010-s003.docx]

| **Authors, Country** | **Title** | **Aim** | **Methods** | **Target Population** | **Demo-graphics mean ± standard deviation** | **Seeking Safety dose mean ± standard deviation** | **Key findings** | **Findings: HCP training/ profession** | **Findings: Professional skills mix delivery sessions** | **Findings: Experience of individuals attending sessions** | **Findings acceptable components** | **Summary** |
| --- | --- | --- | --- | --- | --- | --- | --- | --- | --- | --- | --- | --- |
| Airdrie et al. 2022 (UK) | Investigating the experience of individuals with comorbid posttraumatic stress disorder and substance misuse attending a Seeking Safety group | To evaluate the acceptability and impact of Seeking Safety as a stabilisation treatment | Mixed methods with single-group pre-post-test | Individuals with substance misuse & PTSD | N= 7; female 28.6%; age 29-50 years | Full: 25 sessions, 2-hourly weekly group sessions | Based on Impact of Event Scare-Revised (pre n=7, post n= 3) and Alcohol Use Disorder Identification Test (pre n=5, post n=3). While most made reliable improvement, for two out of three, there was clinically significant change for depression and anxiety. Change was less evident for PTSD. | Psychotherapist | Single facilitator - psycho-therapist | Overarching themes: i) strengthening foundations of the self, ii) understanding of self and role of substances, iii) alternative perspectives, iv) empowerment, agency and activity, v) evocation and management of emotions, vi) safety and validation provided relationally, vii) facilitator as a container | Overarching themes: i) group connection as a source of support and validation, ii) challenges to relational safety, iii) readiness and commitment, iv) preparedness and ability to commit, v) concrete, practical and simple, vi) focusing on the present, vii) consolidation - rather than new content, viii) skill of the facilitator, ix) seeking safety is not an island | Participants of Seeking safety were overwhelmingly positive and reported positive significant changes they had made to their lives. Participants reported achieving a better understanding of themselves and the interaction between mental health and substance use. Participants valued the teaching of concrete, practical and simple skills highlighting grounding as especially helpful. However, an important component appears to be group dynamics and the impact of a facilitator was not there, or there was a second facilitator present, or new members joined. |
| Anderson et al. 2014 (USA) | Does Seeking Safety reduced PTSD symptoms in women receiving physical disability compensation? | To compare PTSD outcomes between dually diagnosed participants with disabilities compared to able bodied participants who received SS or Women's Health Education | Secondary analysis from National Drug Abuse Treatment Clinical Trials Network Women & Trauma Study | Women with disability SS n=12, Women’s health education n=8 | N=20 disability; n=333 no disability. Female 100%, non-Hispanic 95%, Caucasian 50%. Age of women 45.3±6.4 years with a disability, age 38.3±9.3 years with no-disability. | Partial; 12 sessions across 6 weeks of the intervention | Clinical Administered PTSD scores – patients with disability experienced higher decrease in Seeking Safety than Women’s Health Education over 12-month follow-up period, while for non-disabled, similar decrease over time in both SS and Women’s Health Education. | Not noted | Not noted | Not noted | Not noted | After treatment participants were reassessed at week 1, 3-, 6- and 12 months. Pre/posttreatment interviews were completed. Sessions delivered in a group format and included Setting boundaries in relationships, Compassion, Creating meaning, and Asking for help. Women in the Seeking Safety group had lasting reduction in PTSD symptoms when treated with SS but not the Women's Health Education, where PTSD levels returned to baseline at 12 months. However, there are several limitations including a small sample size, the type of disability was not identified. It is unclear whether these findings would be generalisable to men with a disability. |
| Bauer et al. 2022 (USA) | Predictors of Therapeutic Alliance, Treatment Feedback, and Clinical Outcomes among African American Women in Treatment for Co-Occurring PTSD and SUD | To understand and address factors that impede treatment engagement and retentions among black women with PTSD-SUD | Secondary analysis from National Drug Abuse Treatment Clinical Trials Network Women & Trauma Study | Non-Hispanic black women | n=88; female 100%; years in education 12.1±2.09, age 41.1±7.7 | Partial; 12 sessions across 6 weeks of the intervention | Stepwise hierarchical linear regression indicated years of education and previous alcohol/drug treatment attempts were significant predictors early alliance in the second week therapy. Greater education and more treatment attempts were associated with higher early alliance. Alliance did not mediate relationships between these significant predictors and treatment outcomes. | Study therapists were all women; 50% Caucasian, 28% black, 22% Latina. Majority of therapists 56% - had a masters / more advanced degree | Not noted | Educational was a positive predictor of early alliance. These along with other socio-economic factors are important health outcomes for minority and ethnic groups. | Black women who access Seeking Safety treatment are more likely to engage in treatment related tasks and goals more completely, which increased the bond with therapists. | Higher education level predicted greater alliance, controlling for age and income. Greatest number of substance treatment attempts predicted higher earlier alliance. Education level significantly predicted treatment feedback when controlling for alliance. Integration of pre-treatment alliance building strategies with women from ethnic minority groups who have lower levels of education, through directed discussions aiming to develop relationships with therapists, may support better outcomes -especially if there is patient expectation and goal setting increasing the sense of alliance and partnership. |
| Barrett et al. 2015 (Australia) | Treating Comorbid Substance Use and Traumatic Stress among Male Prisoners: A Pilot Study of the Acceptability, Feasibility, and Preliminary Efficacy of Seeking Safety | To examine the acceptability, feasibility, and preliminary efficacy of Seeking Safety | Seeking safety vs. treatment as usual | Men incarcerated within justice system | N=30; male 100%; Age 22–65 years, born in Australian 93.3%. | Partial: 8, weekly individual, 90-min. sessions | Clinician administered PTSD scale and Posttraumatic cognitions inventory scores: reductions in PTSD diagnosis and PTSD symptom severity both study groups at eight-week and six-month follow-ups | Clinical psychologist | Single facilitator; fortnightly supervision for study duration | Client satisfaction scores of seeking safety (n=10) 28.6±4.48/ 32; 90% (n=9/10) would recommend service to others; 80% (n=8/10) reported they would complete the session again as required. | All sessions were useful, giving participants opportunity to reflect on views they held and discuss matters rarely discussed. | Participants of Seeking Safety treatment were supportive of the opportunity. Participants felt longer sessions in the beginning and more sessions 16 vs 8, would have been useful. There was not enough time to discuss matters in details and follow up sessions would have been of benefit. Participants would have liked to have more opportunity to discuss their trauma, and more discussion on anxiety. |
| Boden et al; 2014 (USA) | Coping among military veterans with PTSD in substance use disorder | To examine coping among male military veterans receiving SUD treatment for an alcohol or drug disorder with comorbid PTSD symptomatology | Seeking safety vs. treatment as usual | Military veterans | N=98; male 100%; Age 54±9.6 | Full: 3 sessions pretreatment work focusing on motivational enhancement & encouraging treatment engagement. 25 sessions with twice weekly groups. One topic per session. No supervision, support or training noted. | Zero-order correlation revealed the PTSD symptoms at baseline were positively associated with both avoidance coping (p<0.1), emotional discharge coping (p<0.1) and avoidance coping (p<0.1). Coping scores significantly a) increased for active coping and decreased for b) avoidance, and c) emotional discharge over time. Treatment dose was a significant predictor in the active coping, but not avoidant or emotional coping models. | SS led by Psychologist - PhD level; Treatment as usual led by: therapist, social workers with bachelors or masters level. | Single facilitator twice weekly sessions. PhD level psychologist for SS | Coping measures described with significant changes in i)increased active coping dec, ii) decreased avoidance, iii) emotional discharge coping improved over time. No qualitative findings. | No reflective qualitative findings relative to how individuals found the treatment modality | The study team reported excellent adherence, suggesting participants were engaged in the treatment modality. There were good follow up rates, adherence to the treatment manual in the SS condition, and increased ecological validity resulting from the implementation of the study into a high volume outpatient SUD treatment clinic. |
| Empson et al. 2017 (USA) | Seeking Safety Group Therapy for Co-Occurring Substance Use Disorder and PTSD among Transgender Women Living with HIV: A Pilot Study | To evaluate the acceptability and impact of Seeking Safety in a group of transgender women living with HIV | Evaluative, pre-post-test survey | Transgender women living with HIV | N=7; female 100%; Age 42.37± 10.1 years; Black 71.4% (n=5), Caucasian 28.6% (n=2), 85.7% (n=7) participants reported taking highly active antiretroviral therapy (HAART). | Partial: 12, weekly 120 minute group sessions. 85.7% (n=6/7) participants attended seven or more sessions. | Non significant reduction in mean scores of PTSD Checklist-Civilian Version (p=0.05) and Drug Abuse Screening Test (p=0.06) post-intervention, | None noted | SS led by a registered (licenced) clinical social worker & a Masters prepared social worker | Participants were incentivised to attend $20 for the initial assessment, $180 for completion of all sessions, $120 for 6 - 8 sessions. Both participants and facilitators identified incentives as the most important factor in promoting attendance and retention. Participants found the solidarity and social support aspects of group meetings just as important as therapy. | The group had low levels of literacy, and the flexibility of reducing the number of handouts and promoting group participation through role play improved the quality of each session. | There was a non significant drop in PTSD scores and substance abuse scores. Group facilitators felt the 12 seeking safety modules delivered as 2 hourly sessions provided sufficient time for treatment. Grounding, was the most useful module. Participants felt honesty was felt to be the most problematic. Limitations to the study relate to the small sample size, incentivisation, lack of comparator group or formal assessment of urine toxicology. |
| Holman et al. 2020 (USA) | Quasi‐Experimental Single‐Subject Design: Comparing Seeking Safety and Canine‐Assisted Therapy Interventions Among Mentally Ill Female Inmates. | To determine the effectiveness of two different types of mental health interventions | Quasi-experimental study: Seeking Safety (n=11); Canine assisted therapy (n=8) | Women incarcerated within justice system | N=19; female 100%; Age 24-48 years; Caucasian 78.9% | Partial: 8 weekly group sessions | Percentage of nonoverlapping data (PND) and percentage of data exceeding the median (PEM) was used to assess the efficacy of SS and canine assisted therapy. There was a slight downward trend overall for anxiety, PTSD and depression, but these scores varied from one administration to the next. PEM of anxiety had an effect size of 1.00 and for PND 0.9, indicating treatment is very effective. | The counselors for this group completed training on facilitating the SS intervention, including watching training videos development by Najavits, and reviewing the SS manual. Counselors consulted with a licensed supervising counselor regarding implementation. | Correctional counselors - bachelors degree in criminal justice/ social work. | None noted | None noted | The SS intervention is inexpensive and relatively easy to implement, as it is a manualised treatment protocol. The group can be led by trained paraprofessionals as it focuses on education & skills building. SS does not process trauma or substance use disorder, so additional therapeutic skills by a certified or licensed professional are not required. |
| Kaiser et al. 2015 (Germany) | A Pilot Study of Seeking Safety in a Sample of German Women Outpatients with Substance Dependence and Posttraumatic Stress Disorder. | To evaluate the existing Seeking Safety programs | Evaluative, pre-post-test survey | Women with PTSD and substance use disorder attending outpatients clinics | N= 33; female 100%; Age 40.8±9.6 years; German born 100% | Partial: 12, weekly 90-minute, 10 group and 2 individual sessions | The 17 PTSD measures (the Posttraumatic Diagnostic Scale (PDS), Trauma Symptom Checklist-40 (TSC-40), and Post Traumatic Cognitions Inventory (PTCI)) 14 had effect sizes in the medium to high range, and the other three variables were all just below the medium. From baseline to three-month follow up there were significant improvements PTSD measures of PDS (p<0.0001), TSC-40 (p<0.0001), PTCI (p<0.0001), changes in Alcohol Substance Use index were not significant for drug use (p=0.663) and alcohol use (p=0.015). | Seeking safety training was provided as a 2 day workshops & ongoing supervision from two authors within the team. German translation of the program was completed in collaboration with author of SS | Two clinical psychologists | None noted | End-of-treatment questionnaire indicated a high level of satisfaction. On the scale from - 3 (greatly harmful) to +3 (greatly helpful). Participants reported SS was helpful 2.2±0.9. The highest ratings were found for topics Setting boundaries in relationship 2.5±1.1, Honesty 2.4±1.0, Safety 2.7±1.0, Detaching from emotional pain - grounding 2.6±0.9 | Women who attended at least two individual session and 6 group sessions during the 12 week period of their participation were considered completers. The 12 topics provided in group format included equal amount of behavioural, cognitive and interpersonal topics: Safety, PTSD: Taking back your power, Detaching from emotional pain (grounding), When substances control you, Asking for help, Red and Green flags, Honesty, Recovery thinking, Setting boundaries in relationships, Coping with triggers, Health from anger and self-nurturing. Positive changes were found with regards to PTSD symptoms in those with high adherence. Even with a partial dose patterns were consistent improvement for minimum dose completers. However, there was no improvement with symptoms of substance use disorder. There were limitations to the study with no-intent to treat analysis, no objective measure of substance use, no fidelity monitoring, and lack of statistical power. A substantial subgroup 38% (n=20/33) of the participants did not meet the minimum doses, requiring further research into the reasons for poor treatment compliance. |
| Morgan-Lopez et al. 2013 (USA) | Indirect effects of 12-session Seeking Safety on substance use outcomes: overall and attendance class-specific effects | To examine whether PTSD service as a mediator for the impact of Seeking Safety on in-treatment and post-treatment substance use trajectories, and assess whether mediation effects differ across patient attendance patterns. | RCT: Seeking Safety (n= 176); Women’s Health Education (n= 177) | Women with PTSD and substance use disorder attending outpatients clinics | N=353; female 100%; Age 39.2±9.3 years; Caucasian 45.6%, Black 34% | Partial: 12, 90‐minute twice weekly group sessions for 6 weeks. Adherence was categorised as i) High attendance >90% with 46.3 - 47.9% participants attending; ii) Moderate attendance 50-80% with 46.3 - 47.9% participants attending; iii) low <40% with 24.8 - 26.6% participants attending. | Measures included the use of Post-traumatic stress disorder symptoms scale-self report and Alcohol Substance Index‐Lite. There was a steeper in‐treatment decreases of PTSD symptoms along with the use of alcohol or cocaine in the Seeking Safety (p<0.01) compared to Women’s Health Education group (p<0.05). The mediation effect for Seeking Safety effect on cocaine use as mediated by PTSD Severity was non-significant. | None noted | None noted | PTSD symptoms were assessed at week 1, 3-, 6- and 12months. Groups range from two to eight women, and operated in an open rolling admission (i.e. a participant may enteral the group at session 7 and complete treatment with session 6). Participants remained in the study treatment regardless of attendance unless they missed four consecutive session with no study contact. | None noted | Women with high attendance >90% of Seeking Safety groups had joint in-treatment impact on PTSD and substance use disorder, with a sustained post-treatment changes in PTSD and substance use disorder. Women who attended almost of the available SS sessions showed reduction in alcohol / cocaine use that were transmitted through reduction in PTSD symptoms at 12 months. Those who only managed moderate/ low attendance did not have the same impact on symptoms associated with PTSD and substance use disorders were unchanged. As a result there are likely to be different mediators/ mechanisms of action by which Seeking Safety effects substance use varying across attendance patterns. Future studies should look to record adherence to attendance, and identify reasons impacting moderate/ low attendees. Results from this study suggest that not all patient subgroups benefit from treatment at the same level, and for those who do improve as a result of treatment - different pathways may lead to subsequent improvements in remission of any symptoms. |
| Morgan-Lopez et al. 2014 (USA) | Synergy between Seeking Safety and Twelve-Step Affiliation on Substance Use Outcomes for Women | To explore the additive and interactive effects between Seeking Safety and post-treatment twelve step affiliation and how these effects may vary across treatment attendance patterns. | RCT: Seeking Safety (n= 176); Women’s Health Education (n= 177) | Women with PTSD and substance use disorder attending outpatients clinics | N=353; female 100%; Age 39.2±(9.3 years; Caucasian 45.6%, Black 34% | Patial: 12 sessions, twice weekly groups 120 minutes per session for 6 weeks. At the one week follow up measure asked participants how many times since their last assessment did they attend twelve step or self-help meetings. For the 3-, 6- and 12-month follow up measures - participants were asked how often in the past 30-days had they attended 12-step or self-help meetings. This was then captured as a proportion of possible days meetings were attended. | For high attenders there was significant post-treatment decreases in rate of alcohol use (p<0.001), but not for post-treatment drug use (p=0.93). | None noted | None noted | PTSD symptoms were assessed at week 1, 3-, 6- and 12months. Groups range from two to eight women, and operated in an open rolling admission (i.e. a participant may enteral the group at session 7 and complete treatment with session 6). Participants remained in the study treatment regardless of attendance unless they missed four consecutive session with no study contact. | None noted | This study sought to explore the relationship between extra-treatment recovery support in the form of the 12-step affiliation programme. 30-31% of the group were completers attended 90% of treatment, 27% were titrators achieving 50 - 80% attendance of classes through to the 7th session, and 42-43% were drop-outs and did not attend treatment beyond the 4th session. The results of this study suggest the largest decline in alcohol use rates in women in the Seeking Safety group were those who had the greatest attendance of the 12-step programme. Women who were in the Seeking Safety groups who did not follow up with 12-step programme had worse substance outcomes compared to those in Women’s Health Education. This suggests there may be some synergy between these two programmes and may explain why some women in other studies do poorly. These post-treatment programmes are very accessible, offering social support. However, they are offered in mixed groups with women often only making up 35% of groups. The extent to which mixed groups impacts on women’s entry into this type of post-treatment support is unknown, but is likely to be an important question to explore as part of future work. There are limitations to this work with regards to the lack of intention to treat study design. |
| Najavits et al. 2014 (USA) | Peer-led seeking safety: results of a pilot outcome study with relevance to public health | To evaluate the Seeking Safety model as a peer-led approach | Mixed methods study with pre-post-test | Women with PTSD and substance use disorder in a residential programme | N= 18; female 100%; Age 29.06 ± 7.17 years ; Caucasian 77.8% | Full: 25, twice-weekly, 60-90 min peer-led group sessions | Trauma Symptom Checklist-40 showed significant positive outcomes in trauma-related problems. Effect sizes were large 0.14 for trauma checklist and medium for Basis-32 impulsive-addictive behaviour subscale improved significantly over time. | A clinical staff member straining in SS was present before, during and after groups sessions. However, this was a peer led intervention. | A trained professional. Seven participants served as peer guides in rotating cycles of four weeks each. Criteria for being a peer guide included; having basic stability, attendance of the three hour SS training. | Peer Seeking Safet; 1) There was a guide and co-guide. The guide facilitated the sessions and co-guided assisted by monitoring time, keeping the session true to SS format and watching participants for distress; 2) To prevent unsafe sharing and monopolising time each participant had a maximum of five minutes to speak during sharing; 3) For trauma and substance abuse a 'headlines not details' framework was followed, 4) The list of SS coping skills was displayed during the sessions, 5) Grounding was to be used if participants become distressed, 6) A trained professional would be sought if distress could not be contained, 7) The SS adherence Scale-Brief version was used to record aspects relating to fidelity. | *Thoughts on attending a peer led SS session:* reinforces one addict helping another addict. Empowering for peers. *What did you like about SS?* Goal orientated, positive coping, behaviour modification. *What aspects of SS did you like least?* Long check-in, short check-out. I did not care for the counsellors to sit in. *What changes would you like to see to peer-led SS* - no changes, participants like all aspects including the length of treatment. *How was peer led SS difference from regular treatment? The peer group experience both as guides or participants was the best treatment experience. It is interesting to note this peer group model has been implemented into a clinical pathway which has been sustained, although the co-guide aspect was dropped.* |  |
| Najavits et al. 2016 (USA) | Seeking Safety pilot outcomes at Walter Reed National Military Medical Centre | To evaluate Seeking Safety in military setting providing partial hospital and intensive outpatient levels of care for active duty members of whom 50% had been deployed to areas of active combat. | Treatment as usual plus Seeking Safety | Military veterans with PTSD and substance use disorder | N=24; Male 66.7% Age 28.9±8.77 years. Caucasian 66.75%; 25% African American, 4.1% Hispanic or Latino. 58.3% college educated. | Full: 25 sessions in individual or groups sessions in an open format. Sessions were for 60 - 90 minutes once for individual sessions and twice a week for group sessions. Participants were encouraged to attend as many of the 25 SS treatment topics as possible within their length of stay - typically 3 - 4 weeks. *A priori* 8 sessions of different topics were considered as the minimum dose. Attendance as 11.2±2.9 sessions. Most participants (n=18) completed 9 or more sessions and 6 participants attended the minimum of 8 sessions. | Trauma Symptom Checklist-40 showed significant positive outcomes in trauma-related problems. Effect sizes of change from baseline to end of treatment were large 0.18 for trauma checklist and medium 0.1 for Basis-32 impulsive-addictive behaviour subscale. PTSD/SUD treatment knowledge test for clinicians over time remained unchanged. | Clinicians were trained by a developer-certified SS instructor in a 1-day workshop or by viewing the 4 hours SS training videos, plus 2 hours of phone consultation with the SS instructor. Fidelity was measured using SS Adherence Scale based on session audiotaped. | Ten clinicians conducted Seeking Safety. Social workers (n=7) and n=1 for psychiatry, psychology, recreational therapist and art therapist. 90% were female, with average age of 36.2±11.4 years and clinical experience 10.9±8.6 years. 50% were Caucasian, 40% African American. 60% (n=6) had a Masters degree, 10% (n=1) a MD, 10% (n=1) a doctoral degree. Clinicians reported a mean of 126.7±176 hours (n=9) of conducting Seeking Safety in groups before the study, but with no experience of conducting it as individual sessions. | None noted | Treatment satisfaction were positively rated by participants and clinicians. Client satisfaction scale 1- 4. Participant mean 2.13±0.8; clinician mean 2.3±0.48, with end of treatment participant mean 2.4±0.56 and clinician mean 2.4±0.56. | There were positive results for domains of substance use, PTSD symptoms, functioning psychopathology, and coping. The notable aspects of these results were SS was conducted in a shore time frame averaging 11 sessions. As military hospitals have short lengths of stay PTSD and/ or substance use disorder a model that is feasible to deliver within this time frame is important. |
| Najavits et al. 2018 (USA) | A Randomized Controlled Trial for Veterans with PTSD and Substance Use Disorder: Creating Change versus Seeking Safety | To compare two integrated models for PTSD/SUD: Seeking Safety and Creating Change | RCT - 2 study groups; Seeking Safety (N= 26); Creating Change (N= 26) | Military veterans with PTSD and substance use disorder | N=52; female 26.9%; Age (48.75±10.8 years ); Caucasian 60%, Black 30.1% | Partial: 17, individual weekly 1-hour sessions. Attendance 11.62±5.36. 68% of participants attended the minimum number of sessions. Participants could also attend non-study therapies and support including 12-step self help groups. | Creating Change is a past focused intervention and Seeking Safety a present focused intervention. Intent to treat outcomes for Creating Change versus Seeking Safety found significant effects from baseline to 3 months follow up for PTSD checklist (p<0.005), Current PTSD diagnosis (p<0.005), General self efficacy (p<0.005) and beliefs about substance abuse (p<0.005), Addiction Severity Index (p=0.01), but not for quality of life satisfaction (p=0.38). Patient improved in both treatment options. | Clinicians were trained via in-person training and case supervision. Although training is not required for routine clinical implementation, training and fidelity assessment using developer approved standards are required for research studies that will be published (www.seekingsafety.org -section training) | Five study clinicians (n=4 psychologists, n=1 social worker) 80% female. | None noted | Treatment satisfaction were positively rated by participants with a client satisfaction 1-4 score of 3.6±0.5. Overall helpfulness of treatment was on a scale of 1-7 with 6.73±0.59, for trauma and substance use disorder 6.5±0.74; trauma alone 6.47±0.91, substance use disorder alone 6.33±0.82. | Participants were paid for assessments at baseline, end-of-treatment, and 3-month follow up. They received $40 for each of these assessments and $3 for a weekly urinalysis/ Breathalyzer. Participants with PTSD and substance use disorder improved significantly over time. However, there was no difference in three measures used within the study i.e. coping self efficacy scale, world assumption scale and trauma related guilt inventory. Limitations of the study included the use of highly trained clinicians which may not always be the same in real world setting. The authors of this study reported many questions remain unanswered relative to which clients would most benefit from the treatment? What are the mechanisms of action? What is the emotional intensity of Seeking Safety? What characteristics of therapists and settings might promote the best outcomes? What training is necessary? Are particular treatment topics more relevant than others? Which modality is better individual or group? |
| Najavits et al. 2023 (USA) | A Seeking Safety mobile app for recovery from PTSD and substance use disorder: Results of a randomised controlled trial | To describe the use of a SS app and its performance against a control with regards to primary outcomes of substance use, trauma). | RCT - 2 study groups; N=130, Seeking Safety (N=64); control app (n=66) | Outpatients with PTSD and substance use disorder | N=130; 71.6% (n=83) female, 57.8% Caucasian, 39.7% non-Caucasian | App included: 1) Community of support; 2) Rolling content - each week a new topic launches, 3) Safety orientated features, 4) repeated actions | Attendance at the weekly led peer group Seeking Safety session was very poor with a mean of 1.75 ±1.59 per session. There was no difference between outcomes for the control app and Seeking Safety app, with both conditions of PTSD and substance use disorder significantly improving over time. Effects sizes were low to medium | No training required | None required as mobile application | In the SS arm out of the 64 participants 31% (n=20) were lost to follow up. Of those in the intent to treat arm 60% (n=38) had completion data available | There was very poor attendance of the weekly peer led sessions . Across the 64 sessions there was a mean attendance of 1.75±1.6. End-of-treatment questionnaire indicated a high level of satisfaction. On the scale from - 3 (greatly harmful) to +3 (greatly helpful). Participants reported SS was helpful 1.9 and 1.6 for SS topics. The Mobile App Rating Scale was completed for both SS and the control arm, the scale 1-5 least to most positive, was moderate in both groups SS 3.2±0.3 for SS and control app 3.24±0.3 - suggesting no difference between the apps. Free response positive feedback included - I love the app. I think it can save lives; Negative feedback included; on the SS app focused on technology (slow load, phone incompatibility) and aesthetics. | The SS outperformed the control app with regards to reducing PTSD and substance use disorder, with small to medium effect sizes which was maintain at the 3-month follow up. The low attendance of the peer led SS group may reflect the sample preferred a solitary experience rather than traditional interactive group sessions. Or it could reflect busy schedules or the often chaotic lives associated with PTSD and substance use disorder. Systematic reviews of mental health apps have found there is a short fall in comparison to a control group, especially under more controlled circumstances. Further research is required to better understand what subtypes of people with PTSD and substance use disorder will most likely to benefit from the app. |
| Salvador et al. 2020 (USA) | Impact of Seeking Safety Dose on Depression and PTSD Symptoms Among Pregnant and Post-Partum Women. | To examine the association between the dose of Seeking Safety and PTSD and depression symptom severity | Evaluative, pre-post-test survey | Pregnant and post-partum women residential programme | N=114; female 100%; Age 26.4±5.4 years; Caucasian 49.1% | Full: 25, weekly group sessions, At discharge women in the study had completed an average of 7.5±4.7 sessions. At discharge 64% showed response to treatment compared to baseline and at 6 months 78% (n=39) showed meaningful change. Those with higher number of SS sessions had reduced levels of PTSD. | Post Traumatic Cognitions Inventory: A higher dose of Seeking Safety associated with fewer PTSD symptoms (p<0.05) | Fidelity was assess on a quarterly basis by the clinical supervisor at the agency through the SS adherence Scale. | None noted | None noted | None noted | SS may have the potential to reduce PTSD and depression symptom severity, especially with an increasing number of sessions, although this needs to be confirmed with further research. However, 50% of this group of participants did not complete a 6 month post baseline interview. Those who completed were more likely to have graduated from the programme and to have longer length of stay compared to those lost to follow up 53.2days compared to 38.9 days respectively. In this study there may have been over-representation of women doing better in the community than their counterparts. There is some evidence to suggest those lost to follow up tend to be as healthy as or healthier than those completing follow up interviews. There is also the chance they may not have benefited from SS as a treatment modality. |
| Schäfer et al. 2019 (Germany) | A multisite randomized controlled trial of Seeking Safety vs. Relapse Prevention Training for women with co-occurring posttraumatic stress disorder and substance use disorders | To investigate the efficacy of Seeking Safety compared to treatment as usual and relapse prevention treatment. | RCT: SS + Treatment As Usual (N= 111); Relapse Prevention T +TAU (N= 115); TAU (N=117) | Women outpatients | N=343; female 100%; Age40.9±11.4 years; German-born (90.4%) | Partial: 16, mixed: 2 individual + 14 group 90-minute weekly sessions. Attendance 6.6±5.1. The proportion of participants attending the minimum Seeking Safety dose of 8 sessions was 36.9%. At 6 months 37% (n=41/111). were lost to follow up. | PTSD Symptoms Scale-Interview: there was a significant main effect of time on PTSD severity scores (p=0.001), but no significant effect of group (p=0.748), Baseline-adjusted PTSD severity scores decreased comparably in the three groups. Addiction Severity Index-lite: Alcohol use severity scores were significantly impacted by group (p=0.018), but not by time (p=0.263). | German adaptation of the SS manual (in collaboration with Najavits). Trained by the study team. Group sessions were audiotaped to ensure fidelity. Two in the respective treatments rated adherence to the treatment manual of a randomised selection of 10% of all group sessions. | 39 therapists across all sites - most were psychologist, substance abuse counsellors, psychiatrists, social workers, occupational therapists or nurses | None noted | None noted | Incentives were paid for each study assessment of €20 and €50 depending on the time-point (baseline, post-treatment and follow-up assessments). Most participants were unemployed (77.8%) and had a low monthly income (54.4% of less €1000/month). Seeking Safety + treatment as usual, was as effective as Relapse prevention training + treatment as usual in reducing symptoms of PTSD and substance use disorder. Women who achieved the minimum doses (8 sessions) showed significantly greater reduction in alcohol use compared to treatment as usual alone. 15% of participants dropped out before treatment started. Identifying which participants will benefit most from this type of group work will be important for the sustainability of clinical services in the future. |
| Takahashi, et al. 2022 (USA) | Healing transgender women of colour in Los Angeles: A transgender-centric delivery of Seeking Safety | To analyse the Seeking Safety program’s effectiveness and impact. | Quasi-experimental, single group study | Transgender women | N=81; female 100%; a Age 44.2 ± 12.5 years; Hispanic 70% | Partial: 12 sessions, 90-minuntes, groups (n=10) weekly sessions. Cisgender and transgender groups were initially mixed but as they did not form sufficient cohesion they separated into cis- and transgender groups only. However, in the transgender groups there were tensions relating to age, with older and younger participants often clashing. Seeking Safety counsellors had to act as mediators to bridge the gaps, expanding dialogue and trust to build cohesion. | A significant decrease in the number of days of serious depression 7.26±8.3 vs. 4.56±9 (p<0.05) and anxiety 8.95±9.69 vs 3.95±8.59 in the previous 30 days (p<0.001), along with decreased use of alcohol and illicit substances. | None noted | Counsellor | None noted | None noted | Incentives were paid for each study assessment of $20 non-cash incentives (e.g. gift cards). 78% of the cohort were unemployed, with 26% having less than 11 years of education. From baseline to six months there was significant decreases in the substance use disorder and symptoms of PTSD. |
| Zaccari et al. 2017 (USA) | The participants' voice: A Mixed-Method Evaluation of a Mixed-Gender Seeking Safety Group. | To evaluate Seeking Safety with a mixed-gender group in a residential treatment setting. | Mixed methods, with Pre-Post-test survey | Residential facility | N= 156; female 30.1%; Age 36.6± 10.8 years; Caucasian 69.8% | Partial: 12 sessions, weekly 60-min. group sessions. | Participants who attended at least five modules had lower PTSD civilian checklist scores at 12 weeks (p=0.06). A repeated measure ANOVA showed the main effect was Time but not Gender (p<0.001). | 2-day Seeking Safety fidelity workshop. | Doctoral level psychology intern | Qualitative statements were positive: group participation, setting, motivation/ accountability/ application, awareness, building/ insights gained, therapist, length of session; negative response specific mention of the tools taught, request for more information and nothing new. | Positive correlation found were found with overall helpfulness of the modules. Mean scores ranged from 2.0 for Asking for Help to 2.67 for Health Relationships. | Symptoms of PTSD decreased over time in both men and women. Participants (both men and women) who attended up to 12 modules over 12 weeks showed non-significant greater reduction in PTSD symptoms compared to those who attended 9 or less. There was no difference between male/female outcomes, and that participants were satisfied with the content and format of most modules. Further research is required to explore the use of mixed-gender vs. single gender groups, as well as tracking symptom changes after each group to provide more precise information about the minimum effective dose. Of the 22 modules used in this study participants received a random mix of 12 sessions, making it difficult to interpret what drove changes in symptoms of PTSD and substance use disorder. Some participants of the group sessions were also missing which may have also impacted the results, and there was no control or comparison group, skewing the accuracy of the data and interoperability of the statistical analysis. Future studies will be needed to identify dose, participant cohorts that may benefit from Seeking Safety treatment. |
| PTSD = post-traumatic stress disorder, SUD = substance use disorder, RCT = randomised controlled study | | | | | | | | | | | | |
